# Sequencing of animal viruses: quality data assurance for NGS bioinformatics

**DOI:** 10.1186/s12985-019-1223-8

**Published:** 2019-11-21

**Authors:** Gianpiero Zamperin, Pierrick Lucas, Irene Cano, David Ryder, Miriam Abbadi, David Stone, Argelia Cuenca, Estelle Vigouroux, Yannick Blanchard, Valentina Panzarin

**Affiliations:** 1 Department of Comparative Biomedical Sciences, Istituto Zooprofilattico Sperimentale delle Venezie (IZSVe), viale dell’Università 10, 35120 Legnaro (PD), Italy; 2 French Agency for Food, Environmental and Occupational Health & Safety (ANSES), Ploufragan-Plouzané-Niort Laboratory, Viral Genetics and Biosecurity Unit, 22440 Ploufragan, France; 3 Bretagne Loire University, place Paul Ricoeur CS 54417, 35044 Rennes, France; 4 Centre for Environment, Fisheries and Aquaculture Science (CEFAS), Barrack Road, The Nothe Weymouth, Dorset, DT4 8UB UK; 5 European Union Reference Laboratory for Fish and Crustacean Diseases, DTU aqua, Kemitorvet 202, 2800 Kgs. Lyngby, Denmark; 6 French Agency for Food, Environmental and Occupational Health & Safety (ANSES), Ploufragan-Plouzané-Niort Laboratory, Viral Diseases in Fish Unit, 29280 Plouzané, France

**Keywords:** NGS, Bioinformatics, Proficiency testing, Virology

## Abstract

**Background:**

Next generation sequencing (NGS) is becoming widely used among diagnostics and research laboratories, and nowadays it is applied to a variety of disciplines, including veterinary virology. The NGS workflow comprises several steps, namely sample processing, library preparation, sequencing and primary/secondary/tertiary bioinformatics (BI) analyses. The latter is constituted by a complex process extremely difficult to standardize, due to the variety of tools and metrics available. Thus, it is of the utmost importance to assess the comparability of results obtained through different methods and in different laboratories. To achieve this goal, we have organized a proficiency test focused on the bioinformatics components for the generation of complete genome sequences of salmonid rhabdoviruses.

**Methods:**

Three partners, that performed virus sequencing using different commercial library preparation kits and NGS platforms, gathered together and shared with each other 75 raw datasets which were analyzed separately by the participants to produce a consensus sequence according to their own bioinformatics pipeline. Results were then compared to highlight discrepancies, and a subset of inconsistencies were investigated more in detail.

**Results:**

In total, we observed 526 discrepancies, of which 39.5% were located at genome termini, 14.1% at intergenic regions and 46.4% at coding regions. Among these, 10 SNPs and 99 indels caused changes in the protein products. Overall reproducibility was 99.94%. Based on the analysis of a subset of inconsistencies investigated more in-depth, manual curation appeared the most critical step affecting sequence comparability, suggesting that the harmonization of this phase is crucial to obtain comparable results. The analysis of a calibrator sample allowed assessing BI accuracy, being 99.983%.

**Conclusions:**

We demonstrated the applicability and the usefulness of BI proficiency testing to assure the quality of NGS data, and recommend a wider implementation of such exercises to guarantee sequence data uniformity among different virology laboratories.

## Background

In veterinary medicine, diagnosis, monitoring and prevention of infectious diseases can no longer neglect to perform an accurate genetic characterization of their causative agents [1]. In fact, the number of molecular markers of pathogens with known diagnostic or prognostic value has rapidly increased, allowing a more complete and informative analysis [2–4]. On the other hand, diseases globalization has required diagnostic laboratories to face the challenge of staying at the forefront of research and technology, in order to increase their preparedness in detecting emerging pathogens and new genetic markers responsible for different virulence phenotypes.

In this scenario, the advent of next generation sequencing (NGS) technologies has offered an unprecedented opportunity to generate sequence data in a high-throughput fashion, with or without prior knowledge of the pathogen involved. For these reasons, NGS has become the elected tool for many laboratories performing diagnostic assays, and is being extensively applied to veterinary medicine as well [4, 5]. However, despite the undisputed advantages of this technology, NGS assays involve multifaceted workflows (sample preparation, tissue enrichment, nucleic acid isolation, library preparation, quantitation and pooling, sequencing via unique chemical processes, data analysis) and therefore is potentially prone to the introduction of errors throughout the whole analytical process. In particular, the considerable volume of raw data produced requires complex bioinformatics (BI) analyses that, in turn, necessitate a great amount of computational power in order to extract biological significance from the sequenced samples. For this reason, the BI analysis appears particularly vulnerable and requires appropriate quality checks to ensure the reliability of the results [5]. The steps implicated in this phase can be categorized into three stages: primary, secondary and tertiary analyses [6, 7]. Typically, primary analysis is performed directly in the sequencing platform and involves the conversion of raw signals into base calls and sequence reads. The conversion is carried out by software integrated within the instrument and, if necessary, it is followed by demultiplexing of pooled data, i.e. assignment of reads to the related sample. The output of the machine sequencer is the result of the primary analysis and it is referred to as raw data. Secondary analysis greatly varies in respect to sample type and research aim, and generally comprises a) filtering steps to remove poor data; b) assembly of high quality reads, either by a reference-based or de novo approach; c) variant detection. Tertiary analysis strongly depends on the field of application and consists of human-driven interpretation of sequence data. All these computational steps use filters and quality control (QC) thresholds to convert continuous measurements into discrete results. Notably, the choice of QC metrics can greatly affect the outcome of the BI pipeline, even among laboratories employing the same tools within the same workflow. Additionally, manual curation and interpretation of data at all stages is often implemented, particularly during tertiary analysis, adding another layer of variability. Such diversity and flexibility, both at the computational and interpretation level of the BI analysis, makes it challenging to define universal metrics to assure the quality of sequence data. However, it is of utmost importance to evaluate the performance of post-sequencing analyses as part of the quality management of every laboratory using NGS.

Although exist software programs that allow to perform intra-laboratory quality check for basic issues along different stages of the BI analysis, proficiency testing (PT) remains the most appropriate instrument to assess the comparability of sequence data among different laboratories using diverse analytical methodologies. While PTs are common tools for the evaluation of the wet-bench part, they are still rarely employed for the in silico steps of the NGS analysis, most likely because of the high variability of this phase, the availability of new software and, consequently, the difficulty in establishing standard guidelines [8–10]. In the present study, we conducted for the first time a PT on NGS applied to veterinary virology based on real data, aimed at evaluating the BI pipeline for consensus sequence generation regardless of the techniques adopted for library production and sequencing. This work was carried out within the framework of the Novimark project (Anihwa ERA-Net) (https://www.anihwa-submission-era.net/novimark.html; https://www.izsvenezie.com/novimark-novirhabdoviruses-trouts-salmons/), that aims at identifying virulence markers of two Novirhabdoviruses, the viral haemorrhagic septicaemia virus (VHSV) and the infectious haematopoietic necrosis virus (IHNV), by integrating phenotypic and genetic data related to field strains that originate from different laboratories in Europe. These viruses are of particular concern because they are responsible for two OIE notifiable diseases that severely affect European trout industry, namely VHS and IHN. Fundamental to the Novimark scope was the reliability of viral consensus sequences, as different institutions within the consortium produced genetic data with the NGS technologies and BI pipelines available at their facilities. To reach this goal, sequenced reads generated by three laboratories were gathered together to constitute a unique set of 75 raw data, which was then shared among the participants for BI analysis. The dataset encompassed either VHSV or IHNV field strains as well as one recombinant VHSV. Both viral species have the same genome architecture, constituted by a negative-sense, single-stranded RNA molecule consisting of six genes in the order 3′-N-P-M-G-NV-L-5′. They encode six structural and non-structural proteins, namely the nucleocapsid protein (N), the phosphoprotein (P), the matrix protein (M), the glycoprotein (G), the non-virion protein (NV) and the polymerase protein (L) [11].

The participants were asked to produce a consensus sequence for each strain according to their own BI methodology, and complete genomes were then compared to highlight any discrepancy in terms of type (Single Nucleotide Polymorphisms - SNPs and deletions/insertions - indels) and localization (coding regions, intergenic regions, genome termini). For a randomly selected subset of discrepancies, data were further explored in order to identify analytical steps of the BI pipeline responsible for inconsistent results among laboratories. In this study, we have demonstrated the feasibility of in silico PT applied to veterinary virology and proposed a general flowchart to assess the similarity of outputs from different laboratories using diverse BI pipelines.

## Methods

### Study design

**Fig. 1:**
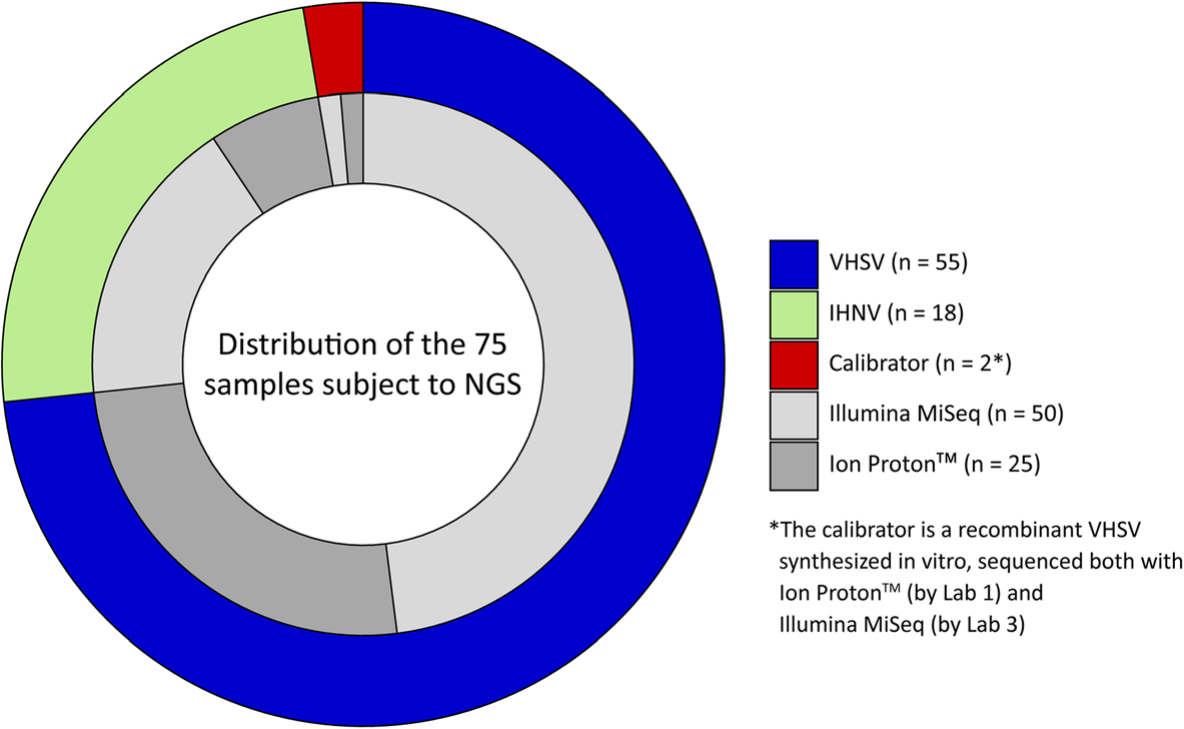
Samples description. Outer and inner rings show samples distribution with respect to viral strain (VHSV, IHNV, calibrator) and to sequencing technology (Illumina MiSeq, Ion Proton™), respectively

Seventy-three VHSV (*n* = 55) and IHNV (*n* = 18) viral strains collected by the Novimark consortium were used for the exercise [Additional file 1] and processed by three participant laboratories, later referred as Lab 1, Lab 2 and Lab 3, according to the protocols and sequencer machines available at their facilities. In detail, 36 VHSV and 13 IHNV were sequenced with Illumina MiSeq by Lab 2 and Lab 3, and 19 VHSV and 5 IHNV were sequenced with Ion Proton™ Sequencer by Lab 1. Additionally, a calibrator specimen constituted by a recombinant VHSV strain (see below) was included in the exercise and analyzed with both sequencing technologies (Fig. 1). All the viruses were subject to library preparation and whole genome sequencing (WGS) according to the protocols reported below. In total, 75 unique raw datasets, later referred as “raw data” or “sample(s)”, were generated by Lab 1, Lab 2 and Lab 3 according to the methodologies implemented at their facilities, and shared in FASTQ format, either as a single file or as two paired files, via a secure File Transfer Protocol (FTP) site. For each sample, participants applied their own BI pipeline and produced a single consensus sequence for comparison.

### Specimens processing and sequencing

#### Lab 1

Viral strains available at Lab 1 repository [Additional file 1], originally lyophilized or frozen at − 80 °C, were inoculated at a multiplicity of infection (MOI) of 2–10 onto 25 cm^2^ flasks seeded with blue gill fry (BF-2) or *epithelioma papulosum cyprini* (EPC) cells [12, 13] grown in Eagle’s minimal essential medium adjusted at pH 7.6 ± 0.2 with 0.19 M Tris-HCl buffer and supplemented with 10% fetal bovine serum (Eurobio), 100 U/mL penicillin and 100 μg/mL streptomycin (Pan Biotech). After 1-h adsorption at 14 °C ± 1, inocula were removed and replaced with fresh medium. Cell cultures were then incubated at 14 °C and checked regularly for the development of cytopathic effect (CPE). Upon completion of CPE, flasks were frozen and thawed, and viral suspensions were collected and clarified at 2000×*g* for 15 min at 5 °C ± 3 °C. Supernatants were then aliquoted and stored at − 80 °C until use.

For each specimen, total RNA was extracted from 1 ml of supernatant using TRIzol™ LS Reagent (Life Technologies) following the manufacturer’s instructions. Double stranded complementary DNA (ds-cDNA) libraries were prepared with the Ion Total RNA-Seq Kit V2 (Life Technologies). The protocol recommended by the supplier was slightly modified and is available from the authors upon request. Finally, cDNA libraries were quality-checked with Agilent 2100 Bioanalyzer (Agilent High Sensitivity DNA kit, Agilent Technologies), quantified by qPCR (Ion Library TaqMan™ Quantitation Kit, ThermoFisher Scientific) and finally sequenced on the Ion Proton™ Sequencer using an Ion PI™ Chip Kit v2 (Life Technologies).

#### Lab 2

Viral stocks provided by the European Union Reference Laboratory for Fish and Crustacean Diseases (Denmark) and processed by Lab 2 [Additional file 1], were freshly propagated at 15 °C on exponentially growing EPC cells [13] cultivated in 75 cm^2^ flasks with L-15 cell medium supplemented with 1 mM L-glutamine, 2% fetal bovine serum, 100 U/ml penicillin and 200 μg/ml streptomycin (Gibco). Once full CPE was obtained, culture supernatants were collected, and cell debris removed by centrifugation at 2500×*g* for 15 min at 4 °C. Viruses were then concentrated by ultracentrifugation at 100000×*g* for 1 h at 4 °C, and pellets re-suspended in 200 μl of cell culture medium.

Viral RNA was extracted using the EZ1 Virus Mini Kit v2.0 and the EZ1 Advanced extraction robot (Qiagen), and eluted in 60 μl of elution buffer. Approximately 100 ng of RNA were reverse transcribed at 37 °C for 1 h in a 20 μl reaction containing 1 mM dNTPs, 500 ng of random primers and 200 units M-MLV Reverse Transcriptase (Promega). Double-stranded cDNA was then generated with random primers using Sequenase V2.0 DNA Polymerase (Affymetrix), purified with the QIAquick PCR Purification Kit (Qiagen) and quantitated using Quantus™ Fluorometer (Promega). Libraries were prepared with the Nextera XT DNA Library Preparation Kit (Illumina), checked for quality and size with Agilent 2100 Bioanalyzer (Agilent High Sensitivity DNA kit, Agilent Technologies) and sequenced with Miseq v2 Reagent Kit (150PE) (Illumina) using Illumina MiSeq platform.

#### Lab 3

Three ml of each virus originating from Lab 3 repository [Additional file 1] were inoculated onto 24-h EPC cells seeded in 72 cm^2^ flasks (Falcon®). After 1-h adsorption at 15 °C under gentle shacking, 15 ml of MEM Eagle (Sigma-Aldrich) supplemented with 10% foetal calf serum (FCS) (Hyclone), 1% L-glutamine 200 mM (Sigma-Aldrich) and 1% antibiotic antimycotic solution 100X (Sigma-Aldrich) were added, and viruses were propagated at 15 °C until completion of CPE. Approximately 20 ml of viral suspension were collected from each flask and clarified at 4 °C for 10 min at 2800×*g*. Subsequently, viruses were concentrated by ultracentrifugation at 90000×*g* for 1 h using a Beckman Coulter Optima L-100 K, and then re-suspended in 500 μl of MEM Eagle (Sigma-Aldrich).

Viral RNA was isolated from 140 μl of concentrated virus using the QIAamp Viral RNA Mini kit (Qiagen) and then subject to retrotranscription with the SuperScript III Reverse Transcriptase (Invitrogen). Double-stranded cDNA was synthesized using NEBNext mRNA Second Strand Synthesis Module (Euroclone), purified with MagSI-NGSPREP PLUS beads (MagnaMedics) and quantified with Qubit dsDNA HS assay kit (ThermoFisher Scientific). The cDNA library was prepared using Illumina Nextera XT DNA Sample Preparation kit (Illumina), and fragments were selected with MagSI-NGSPREP PLUS beads (MagnaMedics). Library was checked for quality and size with Agilent 2100 Bioanalyzer (Agilent High Sensitivity DNA kit, Agilent Technologies) and sequenced with MiSeq v3 Reagent Kit (300PE) using Illumina MiSeq platform.

### Calibrator production and sequencing

The recombinant VHSV strain r23/75 kindly provided by the Institut National de la Recherche Agronomique (INRA) was synthetized via reverse genetics by transfecting EPC cells with an expression plasmid containing the VHSV full-length cDNA [14]. Being a recombinant virus with a predetermined genome sequence, strain r23/75 acted as calibrator for the exercise and was subject to NGS with both Ion Proton™ and Illumina MiSeq according to the procedures available at Lab 1 and Lab 3, respectively. To further verify its sequence, the vector encoding r23/75 genome (p23/75) and used for EPC transfection was also Sanger sequenced by Lab 3. Briefly, the plasmid was propagated in XL 10-Gold ultracompetent cells (Agilent Technologies) and isolated using the EndoFree Plasmid Maxi Kit (Qiagen). The purified vector was then subject to sequencing using primers listed in Additional file 2 and the BrilliantDye™ Terminator Cycle Sequencing Kit (Nimagen) according to the manufacturer’s instructions. Sequencing reactions were then purified with CENTRI-SEP 96 Well Plates (Princeton Separations) and sequenced in a 16-capillary ABI PRISM 3130xl Genetic Analyzer (Applied Biosystems). Sequence data were assembled and edited using the SeqScape software v2.5 (Applied Biosystems) and the final consensus was compared with the genome sequences of r23/75 obtained with NGS. The VHSV complete genome of p23/75 is publicly available under the GenBank acc. no. MK792283.

### Bioinformatics analysis

#### Lab 1

Reads were cleaned with Trimmomatic v0.36 [15] (ILLUMINACLIP: oligos.fasta: 2:30:5:1: true; LEADING: 22; TRAILING: 22 for Ion Proton™ sequencing and 28 for Illumina MiSeq sequencing; MAXINFO: 40:0.2; MINLEN: 36). Bowtie2 v2.2.5 [16] was adopted to perform a rapid alignment with down-sampled reads on the local NT database. VHSV or IHNV complete genomes with the highest number of matching reads were used as a primary reference to align all cleaned reads with BWA v0.7.15 [17]. Based on the alignment, the coverage was estimated using an in-house perl script, bam stat v1.0.13 (http://bamstats.sourceforge.net/) and bam2fastx v1.0 (https://github.com/PacificBiosciences/bam2fastx). Raw reads were down-sampled to fit an estimated global coverage of 80X fold and were de novo assembled with Mira v4.0.2 [18] and, after being cleaned with Trimmomatic, also with SPAdes v3.10.0 [19]. Subsequently, assembled contigs were aligned against the integrated NT database using BLAST [20]. The best match (i.e. the longest sequence corresponding to a complete genome of VHSV or IHNV) was selected as reference for a BWA alignment. The generated SAM file was then used to compute the mpileup using samtools v1.9 [21], and variant calling was performed with bcftools v1.9 [21]. Called variants were then used to produce a consensus sequence with vcfutils.pl v1.9 [21] and seqtk v1.2 (https://github.com/lh3/seqtk). Finally, assembled contigs and BWA alignments were visually inspected for ambiguous bases, indels, ORF integrity, genome integrity and, if required, were manually adjusted.

#### Lab 2

Unless otherwise specified, pre-processing of raw data, reads alignment, variant calling and alignment of the consensus sequences against the reference genomes were done using CLC Genomics Workbench v4.9 (https://www.qiagenbioinformatics.com/). Sequences were trimmed based on quality scores with a cumulative error probability of 0.03, and including a maximum of 3 ambiguous bases. Sequences shorter than 40 bp were discarded. Sequencing adaptors were trimmed with mismatch and insertion costs of 2 and 3, respectively, and with a minimum internal and end scores of 10 and 3, respectively. Reads were aligned against the following reference sequences: VHSV 23–75 (GenBank acc. no. FN665788), VHSV MI03GL (GenBank acc. no. GQ385941), VHSV SE-SVA-14-3D (GenBank acc. no. AB839745) and IHNV (GenBank acc. no. X89213). Mismatch, insertion and deletion costs were set at 2, 3 and 3, respectively. At least half of the reads was required to match the reference genomes with a nucleotide similarity ≥80%. For those reads mapping to multiple positions within the reference genome, the position in the final alignment was randomly assigned. The consensus sequence was inferred based on the alignment with the highest coverage. In case of disagreement among reads, those predominantly represented were used for base calling. Once the drafted consensus sequence was attempted, reads were re-aligned using the same criteria as before. Potentially deleterious mutations were examined by a) aligning the ultimate consensus sequence against the reference with the highest similarity; b) listing any mismatch with a putative detrimental effect on viral phenotype; c) predicting the reading frames using Prokka v1.12 [22]. The support for such deleterious mutations was obtained through examination of the original alignment and with comparison against alternative variant calling tools, such as Snippy [23].

#### Lab 3

Reads quality was assessed using FastQC v0.11.2 (https://www.bioinformatics.babraham.ac.uk/projects/fastqc/). Illumina MiSeq raw data were cleaned by removing: a) reads with more than 10% of undetermined (“N”) bases; b) reads with more than 100 bases with Q score below 10; c) duplicated paired-end reads. Ion Proton™ raw data were cleaned by removing duplicated reads. Filtered reads were clipped from Illumina adaptors Nextera XT or Ion Proton™ adaptors with scythe v0.991 (https://github.com/vsbuffalo/scythe). Low quality ends were trimmed with sickle v1.33 (https://github.com/najoshi/sickle) over a range with minimum average quality of 25 for Illumina MiSeq reads, and 15 for Ion Proton™ reads. Reads shorter than 80 bases or unpaired after previous filters were discarded. Filtered reads were aligned against the integrated NT database (version 8 February 2017) with BLAST v2.6.0+ adopting default parameters and e-value <10e-50. Alignment hits matching against VHSV and IHNV complete genomes, with sequence similarity ≥95% and match length of the query ≥99%, were selected. For each sample, sequences with the highest number of matching reads were chosen as proper reference genomes. Reads were then re-aligned against their respective reference genome selected as above using BWA v0.7.12 and standard parameters. Before SNPs calling, alignments were processed with SAMtools v0.1.19 for conversion into BAM format and sorted by position. Subsequently, potential errors of the alignment were corrected with Picard-tools v2.1.0 (http://broadinstitute.github.io/picard/) and GATK v3.5 [24–26], reads were re-aligned in the proximity of indels and base quality was recalibrated. LoFreq v2.1.2 [27] was then run with the function “--call-indels” to produce a vcf file containing both SNPs and indels. Indels with a frequency lower than 50% and SNPs with a frequency lower than 25% were discarded. Indels within coding regions, determining a reading frameshift, were also removed. The consensus sequence was obtained adopting the following criteria: a) for coverage insufficient for reliable variant calling (<10X), the “N” base was assigned; b) for coverage ≥10X and no SNP call, the reference genome base was assigned; c) for coverage ≥10X and calling of at least one SNP, the nucleotide representing the observed bases was assigned adopting the IUPAC code. High quality reads were re-aligned against their consensus sequence with BWA. Finally, the alignment was visually inspected with tablet v1.14.10.21 [28] and used to manually revise the consensus sequence.

### Consensus sequences comparison

The consensus sequences produced by Lab 1, Lab 2 and Lab 3 were saved in fasta format and collected for subsequent analysis via a secure FTP site. Complete genomes of VHSV and IHNV were aligned separately with MAFFT v7.294b [29] using standard parameters. Differences between the consensus sequences produced by the participants related to the same viral strain were identified with a perl script developed in-house and available upon request. For the recombinant calibrator, consensus sequences obtained with NGS were compared also with the Sanger sequence (Genbank acc. no. MK792283). Discrepancies were evaluated by taking into account their genome localization, namely a) coding regions (CDS), consisting of viral genes translated into viral proteins; b) non-coding intergenic regions, consisting of untranslated sequences that intersperse viral coding regions; c) genome termini, consisting of untranslated regions at the 3′ and 5′ ends of the viral genome. Discrepancies were further categorized as SNPs or indels. A summary of all the inconsistencies observed was generated and scrutinized. PT reproducibility was estimated as the ratio between the number of consistent sites observed and the cumulative genome size of the 75 samples, as previously proposed by Kozyreva et al. (2017) [30]. Accuracy of the BI pipelines was assessed by pairwise comparison of the consensus sequences related to the calibrator sample against its reference genome obtained with Sanger. The accuracy value was estimated as the ratio between observed identical bases and the expected genome size [30].

To deepen the reasons behind inconsistent results, an additional explanatory analysis was performed on a subset of 50 discrepancies randomly selected. We intentionally avoided discrepancies located at genome termini because the variability in the three BI pipelines adopted did not allow a proper assessment of the reasons for variant calls. The analysis was performed in three phases. Firstly, for each discrepancy, participants were asked to provide a justification for their choice in nucleotide assignment. Secondly, all the explanations provided were ascribed to one or a combination of key steps of the BI pipeline namely, i) alignment; ii) variant calling; iii) manual curation; iv) de novo assembly. The three laboratories performed steps i), ii) and iii) in the same sequential order to produce the final consensus sequence. Step iv) was unique to the BI pipeline of Lab 1 and was used during manual inspection of the initial consensus sequence from step iii). Finally, to identify the source of the discrepancies, the explanations of the three laboratories were compared with respect to the order of key steps described within the BI pipeline. Among the three explanations provided for each of the 50 sites, the last by order in the steps series i-iv was arbitrarily assumed to be the source of the inconsistency among laboratories.

## Results

### Comparison of the BI pipelines

In this study, a set of 75 raw data related to 73 fish *Novirhabdoviruses* and one calibrator (Fig. 1) was shared among three laboratories to produce complete genomes according to their own BI pipeline. A flowchart summarizing the different BI pipelines is available as supporting information [Additional file 3]. Lab 1 used a de novo assembly to identify the best available complete genome for a reference-based alignment; the consensus sequence produced was then inspected and manually edited using the initial de novo assembly. Lab 2 used a reference-based assembly on fixed reference sequences to produce an unrefined consensus, utilized as a reference genome for a subsequent assembly. A combination of manual and automatic checks was then performed to ensure the absence of deleterious mutations (i.e. nonsense or indels causing reading frameshift). Lab 3 used a reference-based assembly performed on the most suitable reference sequence previously identified. A final step of manual curation was then performed to ensure that the final consensus obtained was truly representative of the NGS data and to verify the maintenance of the correct reading frame. Undoubtedly, the most heterogeneous steps of the BI pipeline among laboratories were the selection of the reference genome for the generation of the consensus, and its manual curation. The substantial differences in the analytical approach of each participant reflect the great variety of tools and BI pipelines, which in turn impedes the comparison of QC metrics among groups.

### Discrepancies analysis

**Fig. 2:**
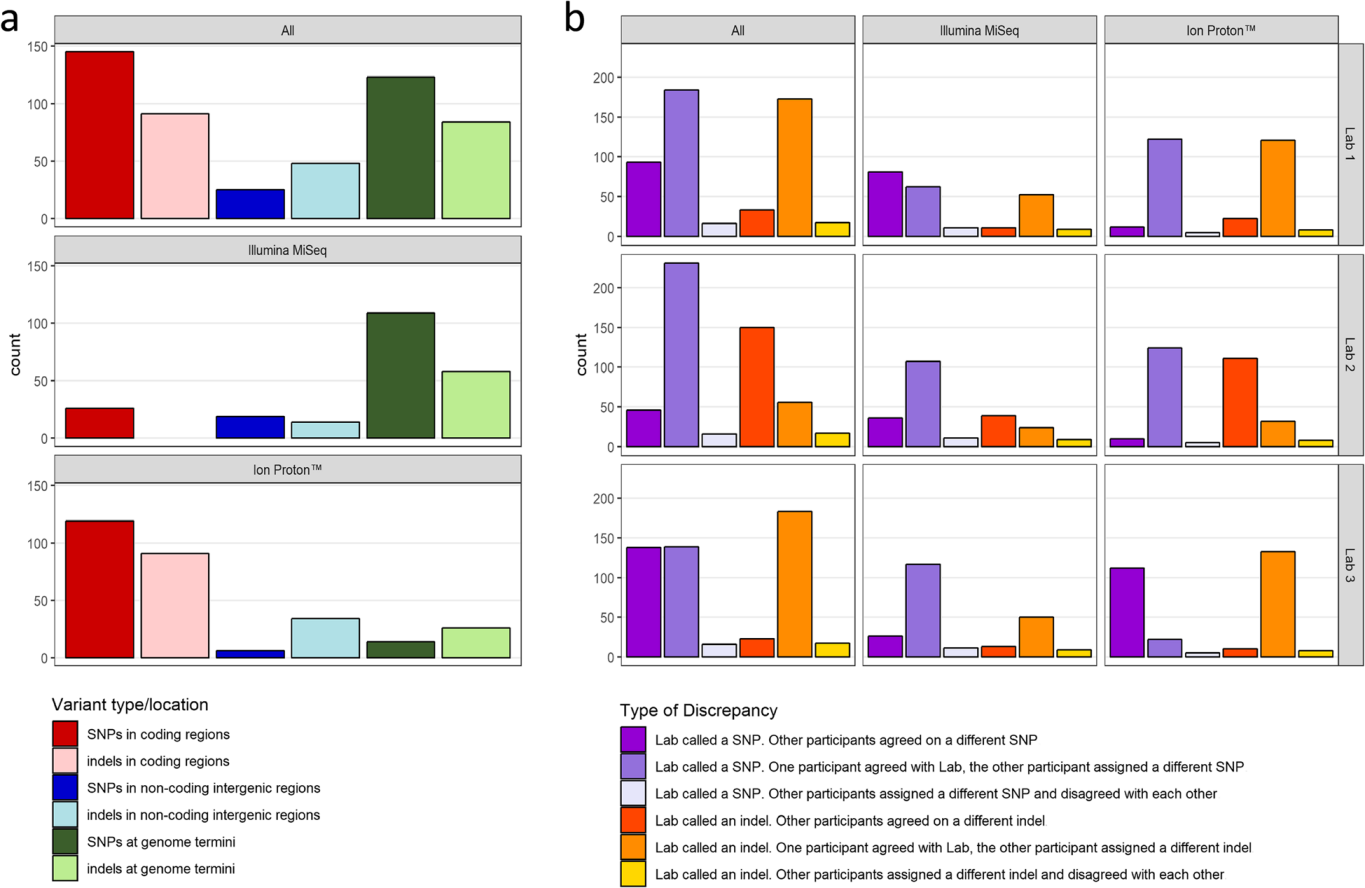
Discrepancies distribution. **a**: Histograms show the total number of discrepancies counted in respect to type (SNP, indel) and genome localization (CDS, non-coding intergenic regions, genome termini); histograms are further divided to display total distribution, and distribution for each type of sequencing technology (Illumina MiSeq, Ion Proton™). **b**: Histograms show discrepancies from the point of view of every participant lab; inconsistent sites are counted in respect to representing the majority or being the odd one out

Consensus sequences obtained from the analysis of 75 raw data were collected, and genomes referring to the same viral strain were compared to highlight any inconsistency. In total, our analysis revealed 526 discrepancies, with an average of 7.0 differences per sample (d/s). A distribution of all discrepancy types occurred, also in respect to sequencing technology, is shown in Fig. 2. A more detailed breakdown of the discrepancies observed in each sample is provided in Additional file 4, where the number of inconsistencies is shown as regards type, genome location, viral species and sequencing technology. The ratio between the total number of discrepant sites and the cumulative genome size for all the raw data was 0.06%. This value was used as a means to evaluate PT reproducibility, corresponding to 99.94%. When considering sequencing technology, our analysis revealed that raw data produced with Illumina MiSeq showed on average 4.5 d/s (227 differences over 50 samples), in contrast to Ion Proton™ that yielded on average 12 d/s (299 differences over 25 samples). Although raw data obtained with Illumina MiSeq appeared to have a smaller number of discrepancies, it is worth noting that different strains were sequenced with diverse systems, thus a direct comparison of the sequencing technology cannot be considered conclusive.

Overall, 39.5% discrepancies among consensus sequences related to the same strain were located at genome termini, 14.1% within intergenic regions and 46.4% affected the CDS. At genome termini, 208 differences were reported, 59.6% of which were SNPs and 40.4% were indels. The majority of these discrepancies were observed for viruses sequenced with Illumina MiSeq (110 SNPs and 58 indels over 50 samples, meaning 2.2 SNPs and 1.2 indels per sample) in respect to Ion Proton™ (14 SNPs and 26 indels over 25 samples, corresponding to 0.6 SNPs and 1.1 indels per sample) (Fig. 2 a). NGS sequencing of genome termini is challenging, both for the wet-bench part and the in silico analysis. Indeed, fragmentation of these portions during library preparation produces fragments with a biased length distribution. Thus, coverage at genome termini shows a declining profile that might dramatically affect the analysis of these regions. Additionally, for reference-based assembly, also the choice of the reference genome is critical for 3′ and 5′ ends reconstruction. For these reasons, we decided to exclude genome termini from the analyses to avoid an excess of complexity in reporting our results and to focus our attention only on SNPs and indels located at coding and intergenic regions. In intergenic regions, 74 differences were reported, 33 for Illumina MiSeq data and 41 for Ion Proton™ data. The majority of the discrepancies (66%) was represented by small indels, with a rate of 14/50 (meaning 14 indels for 50 samples) for Illumina MiSeq data, and 35/25 for Ion Proton™ data. Twenty-five differences were attributable to SNPs, 9 of which involved degenerate base symbols. We performed a more detailed investigation of these latter sites by explicating all possible alternatives for the ambiguous characters detected. Then, for each of the 9 sites involving degenerate base symbols, we checked if at least one alternative was in common between the three labs. We found that, considering degenerations in such a way, all groups identified the same nucleotide. Thus, the discrepancies at these 9 sites were due to different threshold values used for handling degenerate bases rather than differences in the variant calling. At coding regions, the comparison of complete genomes produced with Illumina MiSeq identified 26 SNPs and no indel (Fig. 2 a). The “N” base was assigned to 3/26 SNPs by at least one participant, impeding the unambiguous identification of the nucleotide. Twenty SNPs produced a synonymous codon which resulted in no amino acid change in the related protein. Contrarily, 3 SNPs (corresponding to 1.3% of all the differences observed for samples sequenced with Illumina MiSeq) determined a non-synonymous mutation in the protein product. The analysis of coding regions related to consensus sequences produced with Ion Proton™ revealed a higher number of differences (*n* = 218) (Fig. 2 a), 99 of which were indels and 119 were SNPs. All the indels produced frameshift mutations with the disruption of most of the protein products and the appearance of multiple premature stop codons. Of note, 98 indels were present in the consensus sequences produced by Lab 2 (Fig. 2 b), most likely due to a technical error of the BI pipeline (i.e. variant caller tool). The remaining indel was a 2 bp-long deletion called by Lab 1 on a region with very poor coverage, for which it was very difficult to assess the reliability of the sequenced data. Five SNPs out of 119 involved an “N” base for at least one participant. Seven SNPs, representing 2.4% of all the discrepancies observed in samples sequenced with Ion Proton™, resulted in a missense mutation while 107 SNPs produced a synonymous codon. It is worth noting that approximately 80% (85/107) of the synonymous SNPs are attributable to strain 565-DK-297-HEDEDAM that showed a high number of degenerations within the consensus sequence generated by Lab 3, possibly due to a mixed viral infection. Because this sample accounts for a great amount of discrepancies, we explored the reasons behind this evidence in more depth. A direct inspection of raw data showed that the minority variants frequency for the 85 ambiguous positions ranged between 14.3 and 46.3%. The more conservative approach used by Lab 3 with respect to filtering pipelines, alignments and variant calling might explain the higher number of degenerations present in the complete genome of strain 565-DK-297-HEDEDAM (Figs. 2 b and S2).

Coding regions of the consensus sequences obtained were investigated also for their ability to produce a fully functional protein. For all the samples but one, Lab 1 produced complete genomes fully consistent in respect to CDS length, start/stop codon positions and absence of premature stop codons due to SNPs or indels. The only exception was represented by one sample with a 2 bp indel due to very few reads covering the site, resulting in at least one premature stop codon that disrupts the reading frame. Lab 2 produced identical results when analyzing Illumina MiSeq data; in contrast, the majority of consensus sequences generated from Ion Proton™ data heavily suffered from the presence of indels disrupting the reading frame and introducing multiple premature stop codons (Fig. 2). Lab 3 produced complete genomes fully consistent for all the samples with respect to CDS length, start/stop codon positions and absence of premature stop codons due to SNPs or indels, independently from the sequencing technology.

To better compare the performances of each BI pipeline regardless of previous analytical steps, and assess their accuracy, we sequenced a calibrator specimen constituted by a recombinant VHSV using both the Illumina MiSeq and Ion Proton™ sequencing platforms. Overall, the six genomes produced by the participants were identical, except for 10 discrepancies. One involved a single base located on genome termini where, for Illumina MiSeq data, Labs 1 and 3 called the nucleotide “W” (A/T) and Lab 2 assigned the nucleotide “T”. Nine discrepancies were indels (8 of which located within coding regions and 1 within an intergenic region) present in the consensus sequence produced by Lab 2 based on Ion Proton™ data. This outcome reflects the results obtained by this laboratory for all the other samples analyzed. The agreement between the different BI pipelines was assessed also by comparing the consensus sequences of r23/75 against the sequence of the respective encoding plasmid (p23/75) obtained by Sanger sequencing and used as a gold standard reference. Apart from the 10 discrepancies described above, all the consensus sequences were identical to the reference, suggesting that the BI pipelines used by the participants were basically correct. For Illumina MiSeq data, accuracy of Lab 1 and 3 was 99.991%, while Lab 2 achieved 100% (mean value 99.994%). For Ion Proton™ data, Lab 1 and 3 pipelines were more accurate (100%), while Lab 2 accuracy was 99.919% (mean value 99.973%). Overall accuracy was 99.983%.

### Explanatory analysis of discrepancies

To discover the origin of the inconsistencies among the sequences produced by the participants and referring to the same strain, we performed an explanatory analysis on a selection of 50 discrepancies representative of the observed cases and randomly chosen. The subset included indels and SNPs within intergenic and coding regions. Indels located within the coding regions were excluded from this analysis, as we have previously observed that such inconsistencies are due to a combination of factors, i.e. limits of the Ion Proton™ technology in resolving sequences rich in homopolymers coupled with a technical limitation of Lab 2 BI pipeline probably related to the variant caller adopted.

**Fig. 3:**
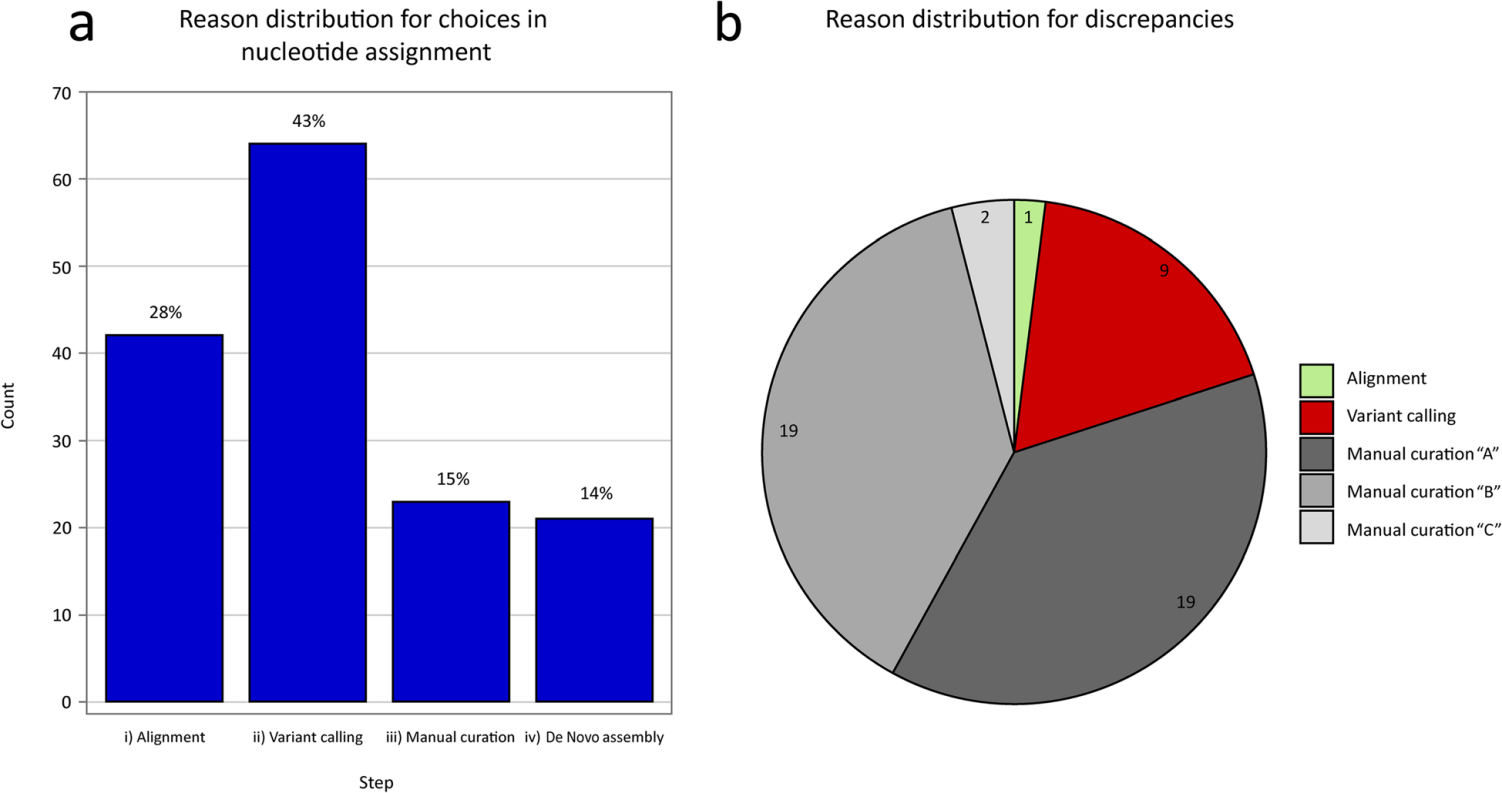
Explanation of inconsistencies. **a**: The histogram shows the total number of justifications for nucleotide assignment given by participants for the subset of 50 discrepancies randomly chosen. Justifications are represented by the BI step to which an explanation is ascribed. **b**: A pie chart showing the source distribution of the discrepancy subset; “manual curation” is further divided into three subgroups, depending from the reason behind the discrepancy itself: “A” arisen when at least one participant assigned a nucleotide during manual curation; “B” arisen after manual curation as well as after de novo assembly during step iv); “C” arisen after de novo assembly of step iv)

When laboratories were questioned individually for the reasons of their choices in nucleotide assignment, 28% were ascribable to alignment, 43% to variant calling, 15% to manual curation and 14% to de novo assembly (Fig. 3 a). However, the comparative analysis of the laboratory scores, aimed at the identification of the cause of the discrepancies, revealed that the majority of them (40 out of 50) were due to manual curation of the consensus sequence (Fig. 3 b). Among these, 19 out of 40 (reported in Fig. 3 b as discrepancies “A”) arose when at least one participant assigned a particular nucleotide during manual curation of step iii). Additional 19/40 discrepancies (reported in Fig. 3 b as discrepancies “B”) occurred after manual curation during step iii) by the three laboratories as well as after de novo assembly by Lab 1 during step iv). Two discrepancies out of 40 (reported in Fig. 3 b as discrepancies “C”) arose after de novo assembly of Lab 1 during step iv). Finally, the remaining 10 discrepancies were due to the variant calling of step ii) performed by the three groups (*n* = 9) or to the alignment of step i) (*n* = 1), which comprises data cleaning, reference choice and alignment tools/QC metrics.

As the manual curation appeared to be the major source for discrepancies, we explored the sites involved and investigated whether such inconsistencies could be resolved with simple adjustments. Twenty-six discrepancies among “A” and “B” involved degenerate bases. In detail, one laboratory reported all the nucleotide variants observed in the consensus sequence, while the other participants selected only one eligible nucleotide. The adoption of a unique threshold value for minimum nucleotide frequency, as well as the setting of common criterion for manual curation of sites where more than one nucleotide is observed, are proposed as possible solutions to obtain consistent results. The two discrepancies “C” were represented by indels found during manual curation of steps iii) and iv). Such indels were missed by one laboratory that did not check the consensus against the raw data during the manual curation step. The implementation of this operation or, alternatively, the adoption of different variant calling tools might be used to solve this type of discrepancy. Finally, inconsistencies among group “A” involving both indels (*n* = 7) and SNPs (*n* = 5) were due to a variety of reasons intrinsic to the different BI pipelines so that no possible shared solution could be proposed to resolve them.

## Discussion

Reliability of viral genomes generated through NGS is of utmost importance both for diagnosticians and researchers aiming to genetically characterize infectious agents. NGS is a complex multifaceted process where quality assurance of sequence data requires a multi-step approach involving both the wet- and the dry-bench parts. Nowadays, PTs of the entire NGS workflow are increasingly being adopted in the clinical diagnostics panorama [8, 31–33]. Importantly, the accomplishment of guidelines established by several institutions and working groups to assure quality standards of NGS data is becoming an important pre-requisite to guarantee laboratory results [7, 34–39].

Although sequence data obtained with NGS are largely used for a variety of applications also in veterinary infectiology [4–10], the implementation of PTs in this field is still at its infancy. In this work, we attempted for the first time to use proficiency testing in veterinary medicine for the generation of consensus sequences of two loss-making salmonid viruses, i.e. VHSV and IHNV, based on real virological data. During this exercise, we assessed the comparability of the BI analysis, regardless of sample preparation procedures, libraries synthesis protocols and sequencing platforms. Indeed, although the evaluation of the whole NGS process by means of PTs has indisputable advantages, challenging individual assay components might be helpful to better highlight critical points of each analytical step [40]. The usefulness of this approach appears clearer when applied to assess the consistency of diagnostic results among institutions that outsource sequencing while performing the BI analysis in-house, or use diverse NGS technologies and BI pipelines. This is the case of the Novimark project, whose ultimate objective is the identification of virulence markers of VHSV and IHNV through WGS. Motivated by the need to rely on fully comparable sequence data generated by different institutions, we organized a proficiency test for BI analysis by sharing the same set of raw data among three laboratories performing NGS, namely Lab 1, 2 and 3. The feasibility of the PT for the evaluation of the post-sequencing analytical steps was first demonstrated by two recent studies applied to oncological diagnosis. Duncavage et al. (2016) [41] used electronic reference data files by editing already existing FASTQ records to introduce variants. In contrast, Davies et al. (2016) [8] used real data obtained from clinical specimens. Both studies evaluated the calling of clinical variants by comparing data provided by laboratories using the same BI pipeline. This impeded the evaluation of results variability deriving from the use of alternative approaches for variant calling, alignment and annotation. In our PT, such a limitation was circumvented by allowing laboratories to adopt their own BI pipeline on a common set of raw data. This enabled to assess the performances of each BI pipeline independently from the wet-bench part of sample preparation, thus permitting the identification of discrepancies at consensus level entirely due to the computational phase. A similar approach has recently been used also by Brinkmann et al. (2019) [10], although in their PT the dataset was artificial and in silico simulated. In our exercise we used real data derived from virus isolates, which realistically represent the potential issues that might arise during BI analysis and add another key point that makes our work unique. However, there are also some limitations to our study. The most significant flaw is likely due to our approach in evaluating the differences between the BI pipelines only at the consensus level. This means that we were actually unable to perform a finer analysis based on the variant allele frequency (VAF) found by each participant, thus underestimating the limitations specific of each BI pipeline. Besides, it is worth mentioning that the assessment of VAF and its implications in the study of viral quasi species would require a much higher sequencing power (i.e. deep sequencing) that we were unable to accomplish in our work. As reviewed in Quiñones-Mateu et al. (2014) [42], this approach is being increasingly used in diagnostic laboratories, given the significance of VAF in human clinical virology [43]. It would be of great interest to implement this practice on a routine basis also for animal viruses, where the selection at viral population level of signatures implicated in immune evasion and host jump are particularly relevant [44–48].

Our data showed that the manual curation of the consensus sequence appeared to account for the majority of inconsistent results among laboratories, while minor reasons were alignment and variant calling. We therefore concluded that manual curation requires harmonization and should be carried out with more attention to ensure the generation of genome sequences fully comparable among laboratories. In particular, as the nucleotide assignment during manual curation might be driven by diverse sources (e.g. re-inspection of raw data, de novo assembly, assessment of the reading frame within coding regions, etc.), it is important to establish common criteria to prioritize the importance of these sources in guiding manual edit. In detail, the adoption of the de novo assembly step and the attribution of ambiguous nucleotides turned out to be the major cause of heterogeneous results. Although we have suggested adjustment strategies to harmonize sequence data (e.g. common threshold values for degenerated nucleotides), the establishment of unique guidelines for BI analysis was far beyond the scope of our work and certainly requires a bigger effort, taking into account also specific purposes and applications of the NGS assay. For the sake of completeness, however, we must recall once again that manual curation is not the only source of discrepancies among laboratories. In fact, indels predicted by Lab 2 when analyzing Ion Proton™ data, as well as SNPs at genome termini observed for Illumina MiSeq data, might be attributable more likely to issues during variant calling rather than manual curation of the final consensus.

Van Borm and collaborators (2016) [9] have recently identified possible sources of error and bias along the NGS workflow. In their review, they have also proposed some primary guiding principles for the analytical validation of NGS methods applied to animal infectious diseases, based on the OIE (World Organization for Animal Health) recommendations [49, 50]. While our exercise as such was not designed to assess assay analytical sensitivity and specificity, we were able to evaluate inter-laboratory reproducibility, defined as the consistency among consensus sequences produced by Lab 1, 2 and 3 under different analytical procedures. In total, the exercise yielded 526 discrepancies, resulting in high concordance among laboratories (99.94% reproducibility). The analysis of a calibrator sample and the comparison of the consensus sequences generated with its reference genome obtained by Sanger allowed us to assess that the BI pipelines were accurate. Overall, reproducibility and accuracy values herein obtained are in agreement with a previous report from Kozyreva et al. (2017) [30], and can be used as a baseline for future PTs on WGS applied to veterinary infectivology.

The sequencing of the calibrator with both Illumina MiSeq and Ion Proton™ enabled us to compare the BI pipelines also in respect to the sequencing technologies, which are both characterized by strengths and weaknesses [51–55]. Ion Proton™ appears more efficient in terms of turnaround time and costs (≤4 h; 80$/Gb) if compared to Illumina MiSeq (24–56 h; 110–212$/Gb). Although both the sequencer machines produce short sequences (≤400 bp), Illumina MiSeq yields high quality reads (0.1% substitution error) of fixed lengths, while Ion Proton™ generates reads within a wide lengths range and of lower quality (1% substitution error). Besides, Ion Proton™ also suffers from an intrinsic high rate of incorrect basecall in homopolymer regions. Despite more reference samples are needed to properly compare the sequencing technologies adopted in this study, our observations confirm that Illumina MiSeq appears more accurate, while indels in homopolymer regions represent a major issue for Ion Proton™.

## Conclusions

In summary, we successfully applied a PT test for NGS BI analysis to animal viruses, which turned out to be valid also outside the human and microbiological clinical context where such exercises have already proved their efficacy. Our results highlight that the manual curation appears the most critical step affecting assay reproducibility, and suggest that a major effort should be made in the future to harmonize analytical steps of the BI pipeline. Indeed, the PT herein presented was useful for the purposes of the Novimark project and, when possible, we recommend implementing ring trials for NGS to guarantee sequence data comparability among different laboratories.

## Supplementary information

**Additional file 1.** List of viruses used for the BI PT. For all the viruses we have reported the laboratory performing wet-bench part, the virial species, and the NGS technology used.

**Additional file 2.** Primers used to Sanger sequence the vector containing the VHSV 23/75 full-length cDNA (p23–75). The nucleotide positions refer to the VSHV sequence under the GenBank acc. no. FN665788.

**Additional file 3.** BI pipelines. Three diagrams show a schematic representation of the BI pipeline for consensus sequence generation adopted in each laboratory.

**Additional file 4.** Distribution of discrepancies per sample. For each raw data, the number of inconsistencies found is expressed as stacked columns. Genome localization is marked with red, blue and green bars (CDS, intergenic regions and genome termini, respectively); SNPs and indels are marked by lighter and darker shades of the corresponding color, respectively. A horizontal double gray bar points the sequencing technology and the viral species for each raw data.

**Additional file 5.** Availability of sequence data. For each sample, we have reported the submitter laboratory, the SRA accession number and the GenBank accession number.

## Data Availability

Illumina MiSeq and Ion Proton™ raw data were submitted to the NCBI Sequence Read Archive (SRA; www.ncbi.nlm.nih.gov/Traces/sra/). We have deposited in GenBank (https://www.ncbi.nlm.nih.gov/genbank/) only the consensus sequences generated by the laboratory performing the sequencing. Consensus sequences generated from laboratories not performing the sequencing are available upon request to the authors. Both SRA and GenBank accession numbers are listed in Additional file 5.

## Abbreviations

BI: Bioinformatics
CDS: Coding sequence
CPE: Cytopathic effect
d/s: Differences per sample
ds-cDNA: Double stranded complementary DNA
EPC: *Epithelioma papulosum cyprini*
FTP: File Transfer Protocol
IHNV: Infectious haematopoietic necrosis virus
Indels: Insertions/deletions
MOI: Multiplicity of infection
NGS: Next generations sequencing
PT: Proficiency test
QC: Quality control
qPCR: Quantitative polymerase chain reaction
RNA: Ribonucleic acid
SNPs: Single nucleotide polymorphisms
SRA: Sequence read archive
VAF: Variant allele frequency
VHSV: Viral haemorrhagic septicaemia virus
WGS: Whole genome sequencing
