# Supplementary material for: Sequencing of animal viruses: quality data assurance for NGS bioinformatics
Source: Virol J. 2019 Nov 21;16:140. doi: 10.1186/s12985-019-1223-8 (PMC6868765; doi:10.1186/s12985-019-1223-8)
Supplement: Supplementary file 2 — Additional file 2. Primers used to Sanger sequence the vector containing the VHSV 23/75 full-length cDNA (p23–75). The nucleotide positions refer to the VSHV sequence under the GenBank acc. no. FN665788. [file 12985_2019_1223_MOESM2_ESM.docx]

| Primer | Sequence 5’🡪 3’ | Position |
| --- | --- | --- |
| VHSSacT7F* | GGATCCCCGCGGTAATACGACTCACTATAGGTATCATAAAAGATGATGAG | - |
| VHSPCR6R* | GTATAGAAAATAATACATACCACAATCG | - |
| VHS_seq_bisM | GTCCGTGCTTCTCTCCTATGTA | 361-382 |
| VHSSeq1 | CAAGTACATCACCAAGAAACTCGG | 607-630 |
| VHS_seq_RbisA | AGTCAGGATGAACGCATAGAGG | 742-763 |
| VHSSeq2 | CAGTGGACTCCCTGGAAGGCCTGGAGG | 1101-1127 |
| VHSSeq3 | CGTCCCTGAAGATCTCCAACTAGACGC | 1674-1700 |
| VHS_seq_bisB | AGCTCCTGAGACGTATCAAGATG | 1718-1740 |
| VHSSeq3bis | AAGGCGGAGCTGGACAAGCTA | 2122-2142 |
| VHS_seq_bisC | GAGAGACGCTCCAGCAGTTA | 2216-2235 |
| VHSSeq4 | GTCCGGTGAGATCTCGCATAGAGGCC | 2603-2628 |
| VHS_seq_bisD | CAGATCTGGAACCTCCTCTGT | 2838-2858 |
| VHSSeq5 | CCCGCTATCAGTCACCAGCGTCTCCG | 3220-3245 |
| VHS_seq_bisE | TGGCCACTACCTGCATAGA | 3256-3274 |
| VHSSeq6 | GATTTCATAGAGGGGGTTTGCACAACC | 3521-3547 |
| VHS_seq_bisF | TGTCAATGCCGATGTCCAGA | 3820-3839 |
| VHSSeq6bis | AGGTGACTGTGACTATGAGGCAG | 4033-4055 |
| VHSSeq7 | GAAACATGGCGACCCAACCCGCGC | 4559-4582 |
| VHSSeq8 | CGGGCGCTTGGCTCCTAAGACAGAGC | 5161-5186 |
| VHSSeq8bis | GTAACATATGTGGAGTACTTGGCC | 5627-5650 |
| VHS_seq_bisN | ACATGTCACCAGATCCTATGAACG | 5932-5955 |
| VHS_seq_bisG | TGACAAACCAAGCGACTCATCT | 6082-6103 |
| VHS_seq_bisH | TGCCCTTTACTCACTCCTAGAG | 6265-6286 |
| VHSSeq9 | CCCAAAATAGGGATATCTGGTACGACGG | 6369-6396 |
| VHSSeq10 | GCCCCGCACGTATGGGCAGATGGAGC | 6968-6993 |
| VHS_seq_bisI | TCGCAGGGAATCCTTCTTCA | 7586-7605 |
| VHSSeq10bis | ACTACGTTACTCACCGGCTCTCGATC | 7758-7783 |
| VHSSeq11 | GGGGGAGACACTCTGGACATGCTCG | 8266-8290 |
| VHS_seq_bisP | TCTGCAGTTGCTCAGCATGA | 8584-8603 |
| VHS_seq_bisO | TGCCCTAGATATTGACGTCAACTC | 8665-8688 |
| VHSSeq12 | GGGAATCACGAAGACTCGGCAGCATCTCCAGG | 8866-8897 |
| VHS_seq_bisJ | AGCGTGTCCAGAGTGTTACA | 8938-8957 |
| VHS_seq_bisK | TCCATTCCTCTCACGATCACTG | 9443-9464 |
| VHSSeq12bis | ACTGGAGTGACCATCCCATTGATGCCA | 9521-9547 |
| VHS_seq_bisL | CTCATTGTGGTGATCGGAGGA | 9899-9919 |
| VHSSeq13 | CACGGTCAAGGGAAACAGGGACCTCC | 10111-10136 |
| VHSSeq14 | CTCCTCGGATGCCATTACCGAGAAGGG | 10720-10746 |

*Primers hybridizing within the plasmid sequence
