# Supplementary figures and images for: Sequencing of animal viruses: quality data assurance for NGS bioinformatics
Source: Virol J. 2019 Nov 21;16:140. doi: 10.1186/s12985-019-1223-8 (PMC6868765; doi:10.1186/s12985-019-1223-8)

# Lab 1 BI pipeline

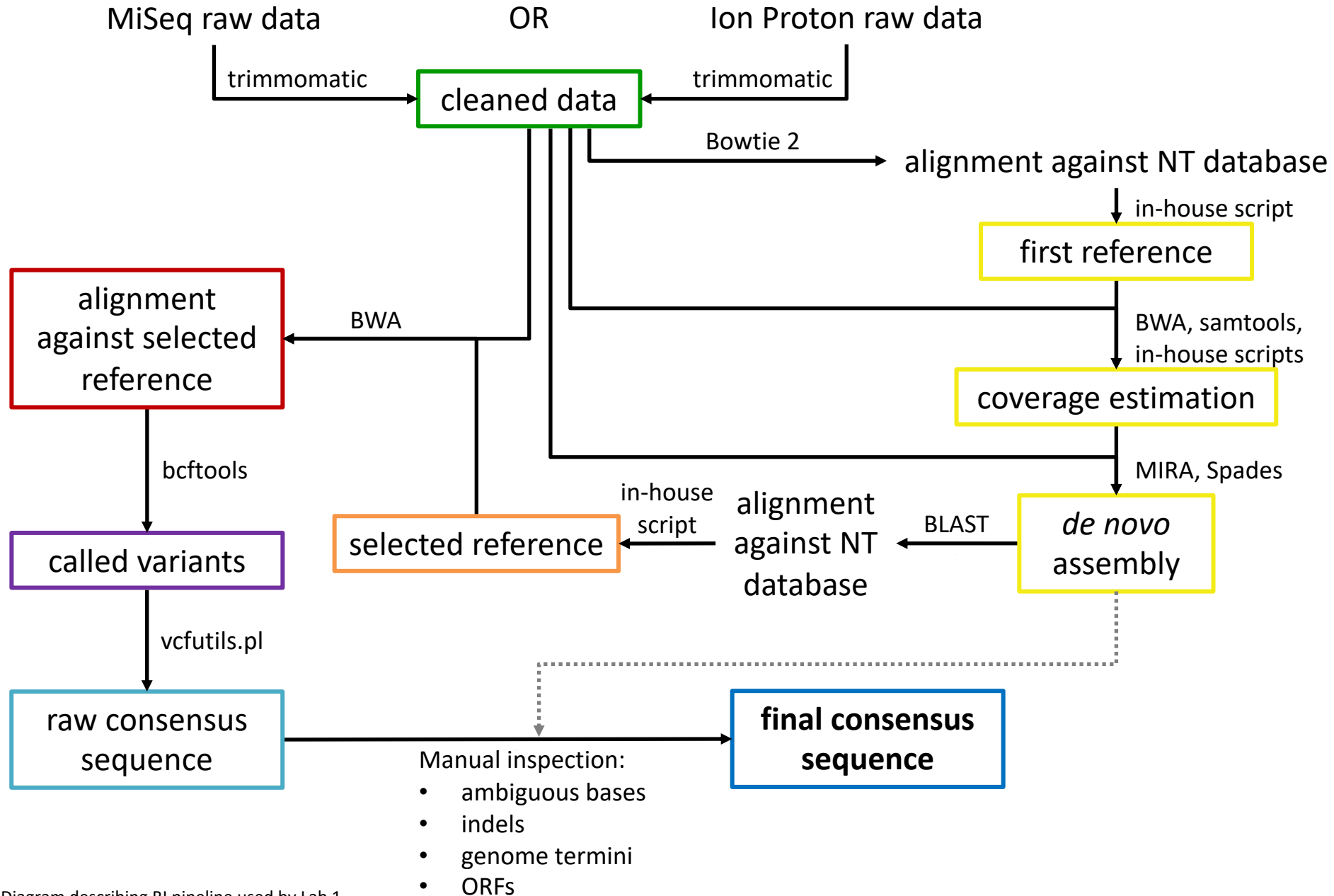

Diagram describing BI pipeline used by Lab 1.

# Lab 2 BI pipeline

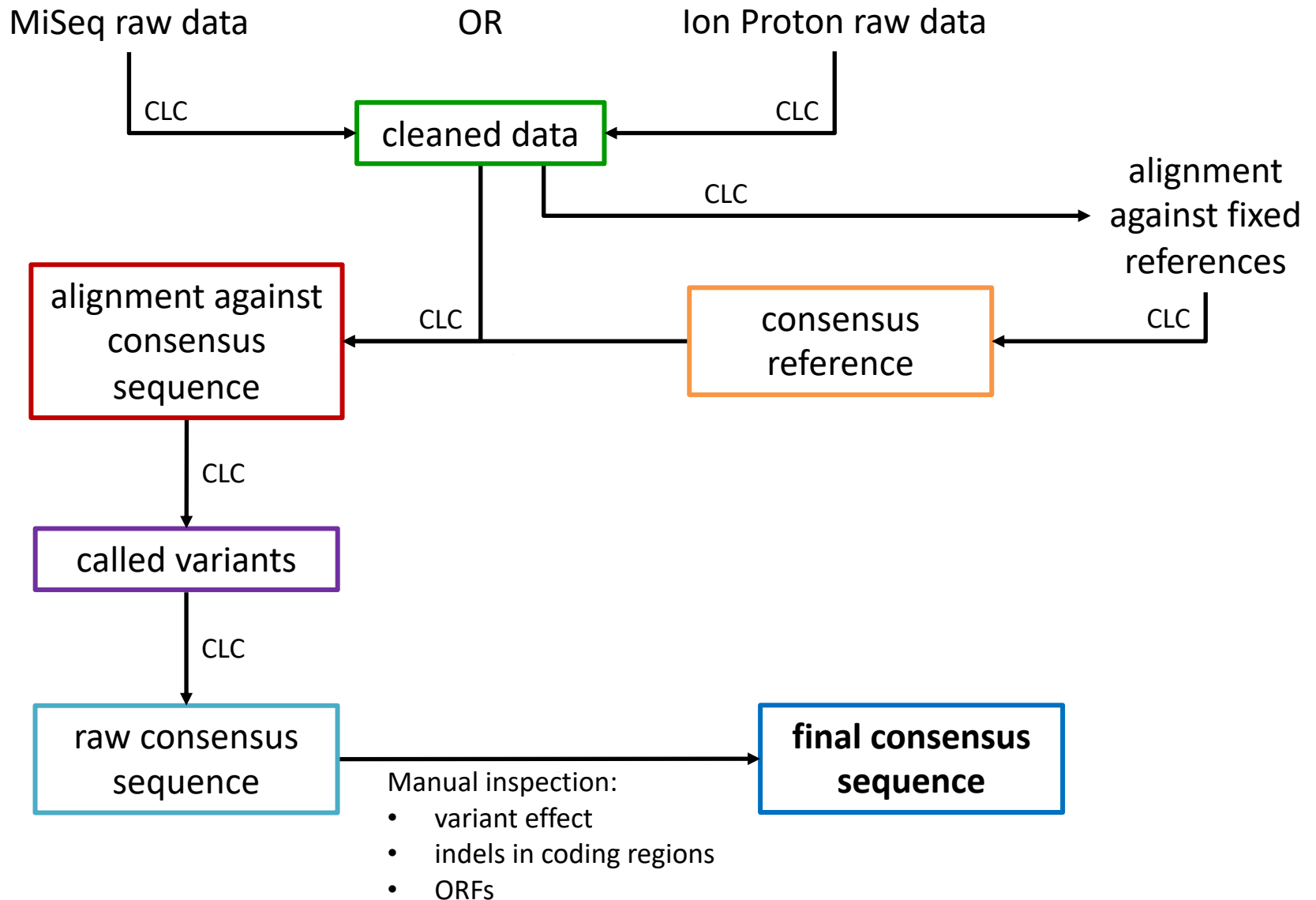

# Lab 3 BI pipeline

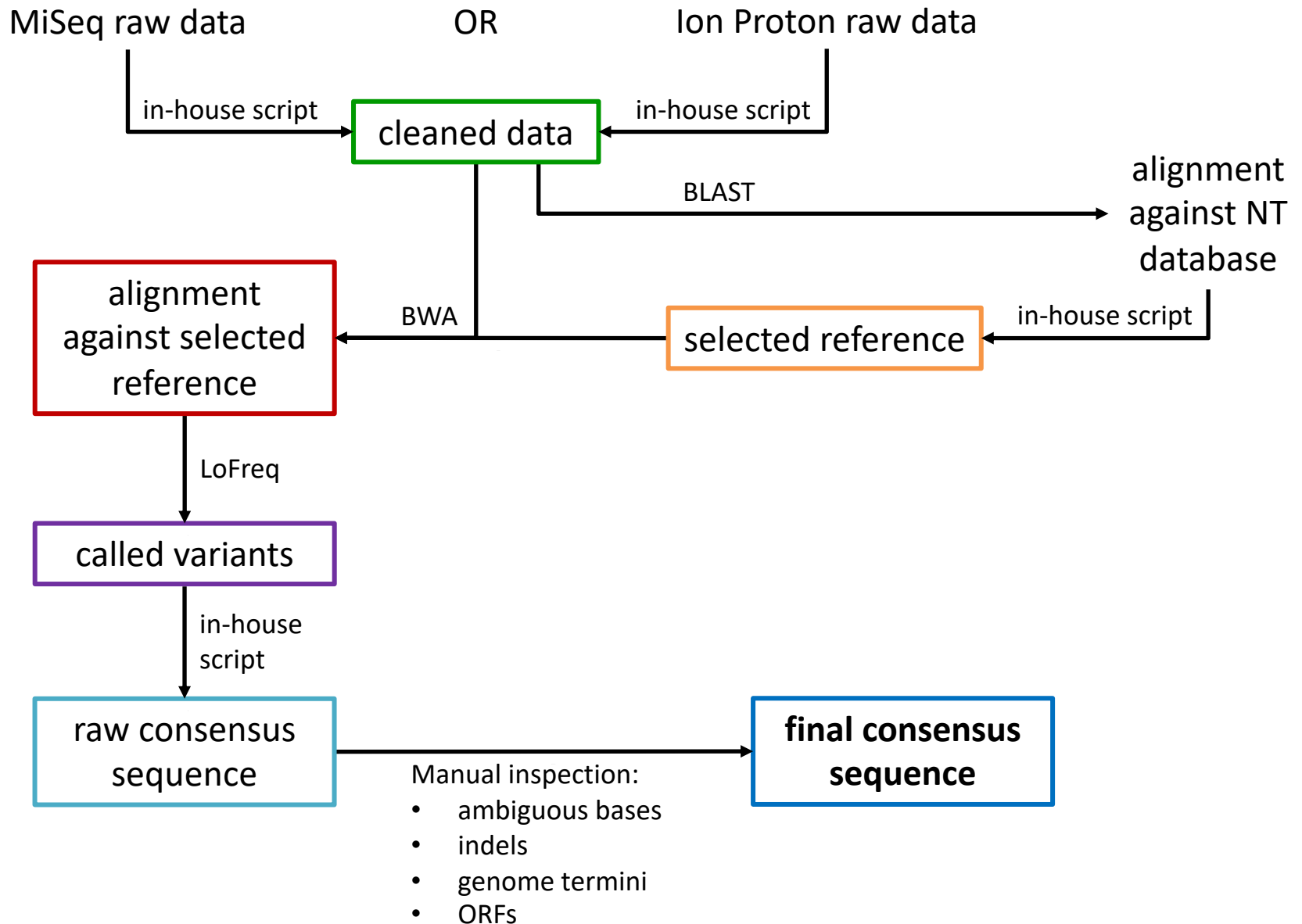

Supplement: Supplementary file 3 — Additional file 3. BI pipelines. Three diagrams show a schematic representation of the BI pipeline for consensus sequence generation adopted in each laboratory. [file 12985_2019_1223_MOESM3_ESM.pdf]

Distribution of discrepancies among samples

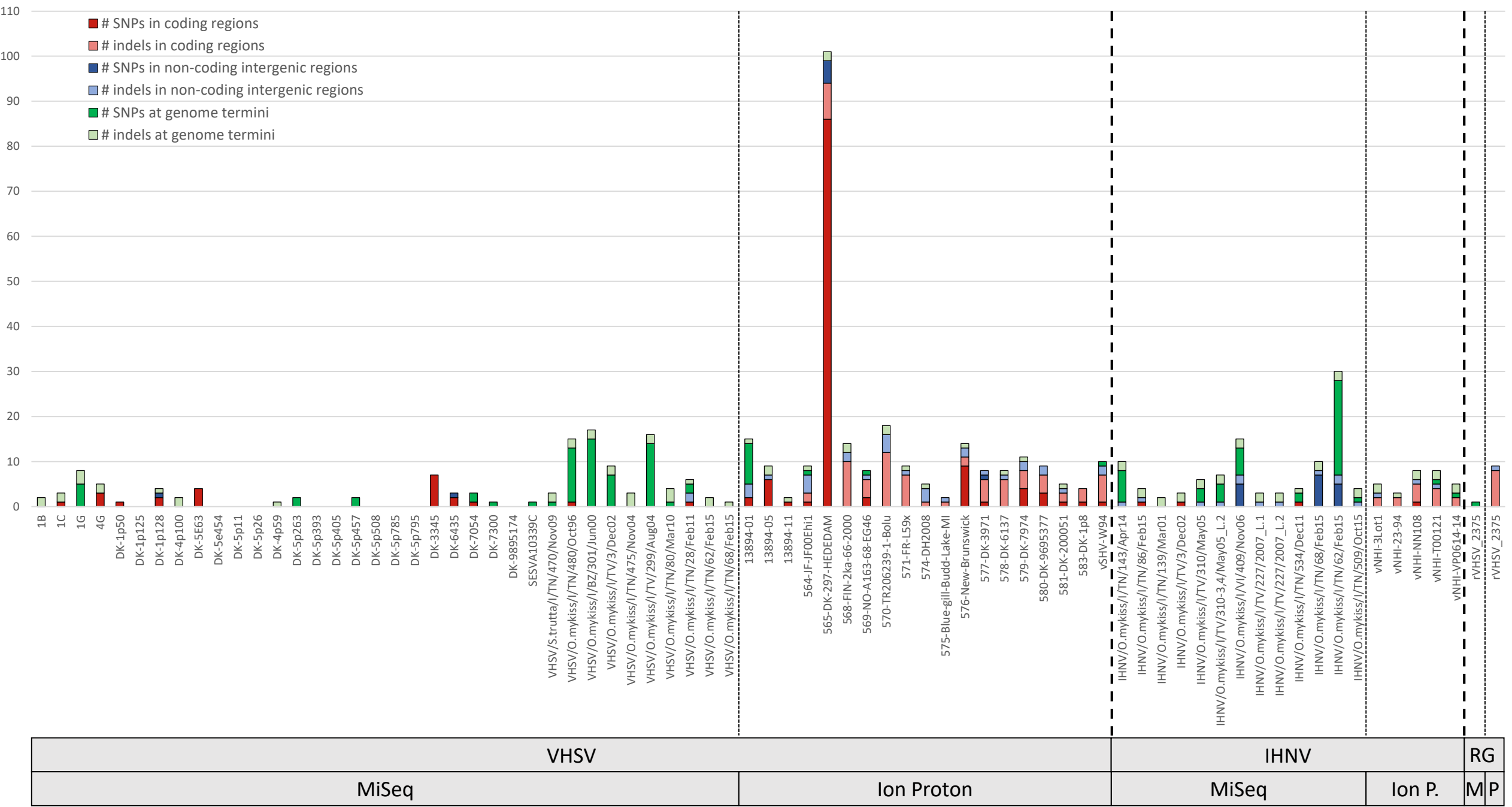

Supplement: Supplementary file 4 — Additional file 4. Distribution of discrepancies per sample. For each raw data, the number of inconsistencies found is expressed as stacked columns. Genome localization is marked with red, blue and green bars (CDS, intergenic regions and genome termini, respectively); SNPs and indels are marked by lighter and darker shades of the corresponding color, respectively. A horizontal double gray bar points the sequencing technology and the viral species for each raw data. [file 12985_2019_1223_MOESM4_ESM.pdf]
